# ASRGL1 Correlates With Immune Cell Infiltration in Hepatocellular Carcinoma and Can Serve as a Prognostic Biomarker

**DOI:** 10.3389/fonc.2021.680070

**Published:** 2021-06-25

**Authors:** Cailin Xue, Peng Gao, Xiaohan Cui, Xudong Zhang, Jin Lei, Renzhi Li, Chunfu Zhu, Xihu Qin

**Affiliations:** Department of Hepatobiliary Surgery, The Affiliated Changzhou No. 2 People’s Hospital of Nanjing Medical University, Changzhou, China

**Keywords:** HCC, ASRGL1, biomarker, immune, immune cell infiltration

## Abstract

**Background:**

The enzyme L-asparaginase (ASRGL1) catalyzes the hydrolysis of L-asparagine (Asn) to L-aspartic acid (Asp) and ammonia. Numerous studies have shown a strong correlation between ASRGL1 expression and tumorigenesis. However, the expression and biological function of ASRGL1 in hepatocellular carcinoma (HCC) are still unclear.

**Methods:**

We explored the mRNA expression of ASRGL1 in HCC using the HCCDB, Oncomine, and TIMER 2.0 databases. Western blotting and immunohistochemical analyses were also used to determine the mRNA expression of ASRGL1 in HCC. LinkedOmics was used to analyze the genes co-expressed with ASRGL1 and regulators including kinases, miRNAs, and transcription factors. The Gene Ontology (GO) terms and Kyoto Encyclopedia of Genes and Genomes (KEGG) pathways of the co-expressed genes were also investigated using LinkedOmics. The correlation between ASRGL1 expression and immune infiltrates was analyzed using the TIMER 2.0 and Gene Expression Profiling Interactive Analysis (GEPIA) databases. The effects of ASRGL1 expression on patient outcome were investigated using the UALCAN and GEPIA databases, and the Kaplan–Meier plotter. c-Bioportal was used to explore the mutations of ASRGL1 in HCC.

**Results:**

Compared with the adjacent tissues, ASRGL1 was upregulated in HCC. High ASRGL1 expression in HCC indicated poor relapse-free survival, progression-free survival, disease-specific survival, and overall survival. The expression of ASRGL1 was significantly correlated with infiltrating levels of B cells, CD4+ T cells, macrophages, neutrophils, and dendritic cells in HCC.

**Conclusion:**

Our findings suggest that ASRGL1 is overexpressed in HCC and that ASRGL1 expression was significantly correlated with immune infiltration in HCC and prognosis. Therefore, ASRGL1 may serve as a biomarker for the early diagnosis and treatment of HCC.

## Introduction

Hepatocellular carcinoma (HCC) is the fourth most common digestive system malignancy and approximately 42,030 new cases of HCC are diagnosed each year (1). At present, surgery is the first-line treatment for HCC. Owing to the complexities associated with early diagnosis, the relative ease of intrahepatic metastasis and the high postoperative recurrence rate, the 5-year survival rate for patients with HCC is only 40%, which is much lower than other digestive malignancies (2). Although many molecular targeted drugs, such as sorafenib and regorafenib, have been used for inoperable and postoperatively recurrent HCC, the clinical treatments for HCC are unsatisfactory (3). Therefore, it is essential to uncover the specific molecular mechanism underlying the pathogenesis and metastasis of HCC.

Metabolic abnormalities play important roles in the occurrence and progression of tumors (4). In this study, we have focused on the enzyme L-asparaginase (ASRGL1), which catalyzes the hydrolysis of L-asparagine (Asn) to L-aspartic acid (Asp) and ammonia (5). The abnormal expression of ASRGL1 has been found in breast, ovarian, and prostate cancers (6–9). In breast cancer, the strong expression of ASRGL1 in tumors can promote proliferation and inhibit apoptosis in cancer cells (9, 10). ASRGL1 is considered as a biomarker of endometrial tumors and low ASRGL1 expression is associated with a poor outcome of the cancer (7, 11). ASRGL1 has been used to treat hematological malignancies and has a good clinical therapeutic effect (12). However, the clinical significance of ASRGL1 in HCC is still unclear.

In this study, by using databases including The Cancer Genome Atlas (TCGA), Oncomine, and TIMER 2.0, we investigated the network of co-expressed genes and potential function of ASRGL1. The results showed that ASRGL1 was overexpressed in HCC and had a negative correlation with patient outcome. Moreover, ASRGL1 may participate in immune infiltration and promote the progression of HCC. Overall, our research revealed a new perspective on the progression of HCC and provided a potential target molecule for the treatment of HCC.

## Materials and Methods

### HCCDB Database Analysis

The HCCDB database is a public HCC gene expression profiling database that contains 3,917 samples from the Gene Expression Omnibus (GEO) and TCGA database (13). In this study, the HCCDB database was used to investigate the expression of ASRGL1 in HCC.

### Ethical Approval and Consent to Participate

Liver cancer tissues and the corresponding adjacent tissues were collected from Nanjing Drum Tower Hospital from 2018 to 2020 and approved by the Committee for Ethical Review of Research. Written informed consent regarding tissue collection was obtained from all patients.

### Cell Culture and Clinical Samples

The cell lines used in this study, including LO2, SMMC-7721, Hep3B, HepG2, 97H, 97L, HCC-LM3, and Huh7 were obtained from the ATCC. The cells were cultured in DMEM supplemented with 10% FBS, 100 mg/l streptomycin, and 10^5^ U/L penicillin. The cells were incubated in a 5% CO_2_-humidified incubator at 37°C. Liver cancer tissues and the corresponding adjacent healthy tissues were collected from patients treated at Nanjing Drum Tower Hospital between 2018 and 2020. The tissues were stored in liquid nitrogen until use. Before sample collection, written informed consent collection was obtained from all patients.

### Western Blotting

Proteins were extracted from cells and tissues using a radioimmunoprecipitation assay kit (Beyotime, Shanghai, China) supplemented with 0.1% protease inhibitors and 0.1% phosphorylase inhibitors. The protein concentration was quantified using the BCA Protein Assay Kit (Beyotime, Shanghai, China). Subsequently, the protein samples were separated on 10% SDS-PAGE gels, and the separated proteins were transferred to a PVDF membrane (Millipore). The membranes were incubated in 5% milk for 2 h at room temperature to block non-specific binding, and then incubated with appropriate antibodies overnight at 4°C. The following primary antibodies were used: ASRGL1 (#11400-1-AP, 1:1,000, Proteintech), GAPDH (#5174, 1:1,000, CST). The membranes were visualized with an ECL detection system after incubation with secondary antibody at room temperature for 2 h.

### Oncomine Database Analysis

Oncomine is a gene expression microarray database for visualized analysis of tumor data (https://oncomine.org/resource/login.html) (14). In this study, Oncomine was also used to explore the mRNA expression of ASRGL1 in HCC. The thresholds were as follows: P-value, 0.05; fold-change, 1.5; gene ranking, all.

### UALCAN Database Analysis

UALCAN (http://ualcan.path.uab.edu/), a website based on TCGA, can be used to analyze the relative gene expression in tumor and normal tissues, as well as the relative gene expression based on race, tumor grade, and other clinicopathological features (15). In this research, this database was employed to explore the mRNA expression of ASRGL1 across the tumor grades.

### GEPIA Database and Kaplan–Meier Plotter Database Analysis

GEPIA (http://gepia.cancer-pku.cn/) is an interactive online web tool for the analysis of the tissue samples from the TCGA and GTEx projects (16). The Kaplan–Meier plotter database (http://kmplot.com/analysis/) provides data analysis of 10,461 samples, including samples of gastric cancer, breast cancer, and HCC (17). In this study, the GEPIA and Kaplan–Meier plotter databases were used to explore the association between ASRGL1 and survival outcome of patients with HCC.

### c-BioPortal Database Analysis

c-BioPortal (http://cbioportal.org) is an open-access online database providing multidimensional analysis of cancer genomics data sets. The multidimensional analysis usually includes mutation, copy number variation (CNV), and gene co-occurrence. In this study, c-BioPortal was used to investigate ASRGL1 mutations and CNV in HCC (18).

### TIMER Database Analysis

TIMER is a comprehensive resource that provides systematic analysis of the immune infiltrates in 32 types of cancers (https://cistrome.shinyapps.io/timer/) from TCGA (19). The TIMER database applies a deconvolution method to investigate the abundance of tumor-infiltrating immune cells (TIICs) based on the gene expression profiles. In this research, we investigated the correlation between the mRNA expression of ASRGL1 and tumor-infiltrating immune cells, including B cells, CD4+ T cells, T cells (general), neutrophils, macrophages, dendritic cells (DCs), and natural killer (NK), cells in HCC and stomach adenocarcinoma (STAD). We also explored the relationship between the gene markers of TIICs and the expression of ASRGL1 using TIMER. Gene markers for B cells, T cells, TAMs, macrophages, monocytes, neutrophils, NK cells, DCs, exhausted T cells, and Treg cells were selected based on the previous research (20).

## Results

### ASRGL1 Was Upregulated in HCC

**Figure 1:**
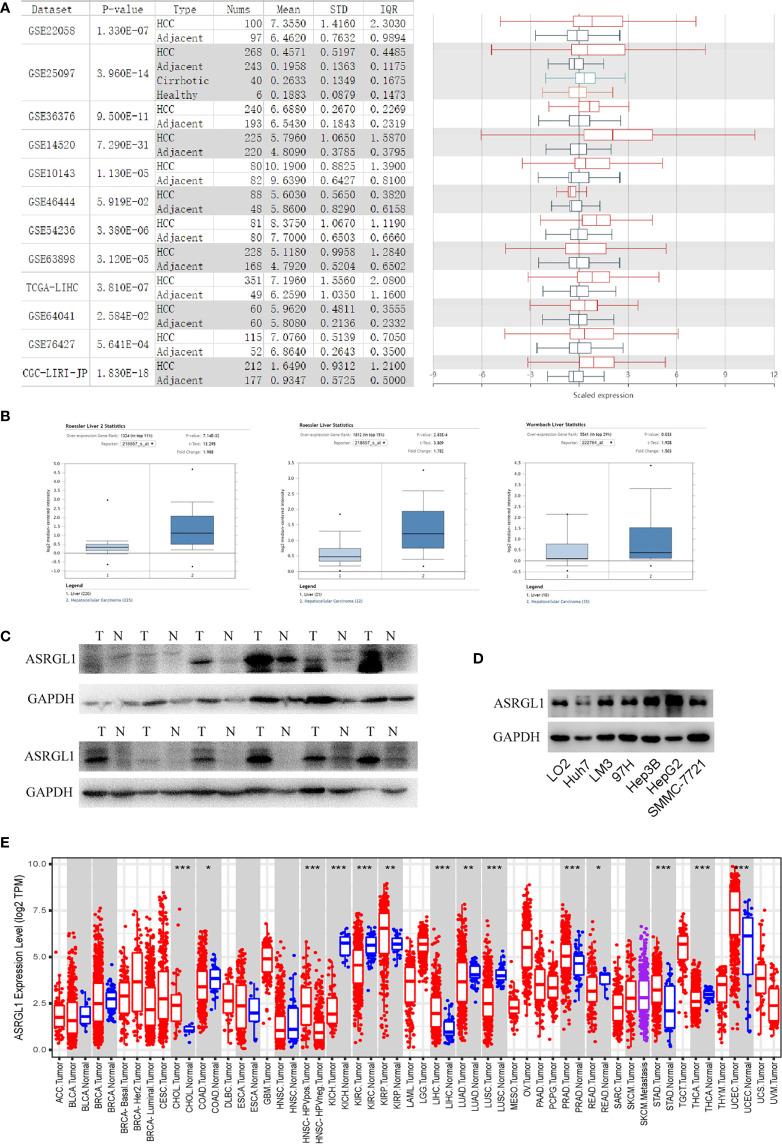
The expression of ASRGL1 in HCC. **(A)** The graph and the corresponding plot show the mRNA expression of ASRGL1 in tumor tissues and adjacent healthy tissues, according to the *t*-test in HCCDB. **(B)** Box plot showing ASRGL1 mRNA expression in the Roessler Liver, Roessler Liver 2, and Wurmbach Liver datasets, respectively. **(C, D)** Western blot showing that ASRGL1 was upregulated in HCC tissues and HCC cell lines compared with adjacent healthy tissues and LO2 cells. **(E)** Expression of ASRGL1 in different tumor types from the TCGA database in TIMER. *p < 0.05, **P < 0.01, ***P < 0.001. (ACC, BLCA, CESC, CHOL, COAD, DLBC, ESCA, GBM, HNSC, KICH, KIRC, KIRP, LAML, LGG, LIHC, LUAD, LUSC, MESO, OV, PAAD, PCPG, PRAD, READ, SARC, SKCM, SKCM, TGCT, HCA, THYM, UCEC, UCS, UVM).

**Figure 2:**
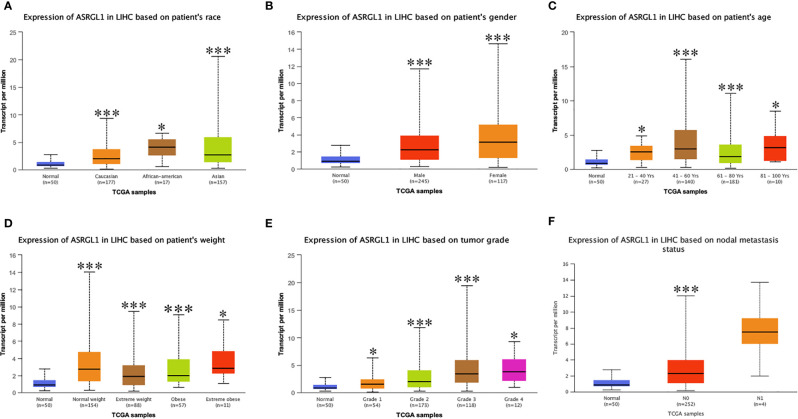
The mRNA expression of ASRGL1 in subgroups of patients with HCC, stratified based on sex, age, and other criteria (UALCAN). Box-whisker plots showing the expression of ASRGL1 in subgroups of LIHC samples. **(A)** The mRNA expression of ASRGL1 in healthy and HCC samples. **(B)** The mRNA expression of ASRGL1 in healthy individuals of either sex and in male or female patients with LIHC, respectively. **(C)** The relative mRNA expression of ASRGL1 in normal individuals of any age or in patients with LIHC between 21 and 40, 41 and 60, 61 and 80, and 81 and 100 years of age. **(D)** The relative expression of ASRGL1 in healthy” patients subjects and African American, Caucasian, and Asian patients with HCC. **(E)** The mRNA expression of ASRGL1 in healthy individuals in patients with stages 1, 2, 3, or 4 HCC. **(F)** The mRNA expression of ASRGL1 in healthy individuals or patients with grade 1, 2, 3, or 4 HCC tumors (central mark: the median; edges of the box: the 25^th^ and 75^th^ percentiles). The *t*-test was used to estimate the significance of differences in gene expression levels between groups. *p < 0.05; **p < 0.01; ***p < 0.001.

HCCDB and Oncomine were used to investigate the expression of ASRGL1 in HCC. We found the ASRGL1 mRNA in HCC was significantly higher than that in the adjacent tissues based on the HCCDB database and Oncomine (**Figures 1A, B**). To verify the results in the database, we further explored the expression of ASRGL1 in liver cancer and HCC cell lines; we found that the expression of ASRGL1 in cancer tissues was significantly higher than that in paracancerous tissues (**Figure 1C**) and that the expression of ASRGL1 in liver cancer cells was higher than that in LO2 liver cells (**Figure 1D**). According to the TIMER 2.0 database, ASRGL1 mRNA is strongly expressed in a variety of cancers, including cholangiocarcinoma, pancreatic cancer, and HCC (**Figure 1E**). Subsequently, using UALCAN, the expression of ASRGL1 was found to be correlated significantly with race, age, sex, body weight, tumor grade, and nodal metastasis status (**Figure 2**). The above results all indicated that ASRGL1 is abnormally expressed in HCC and may serve as a biomarker of HCC.

### ASRGL1 Expression Was Positively Correlated With Patient Outcome

**Figure 3:**
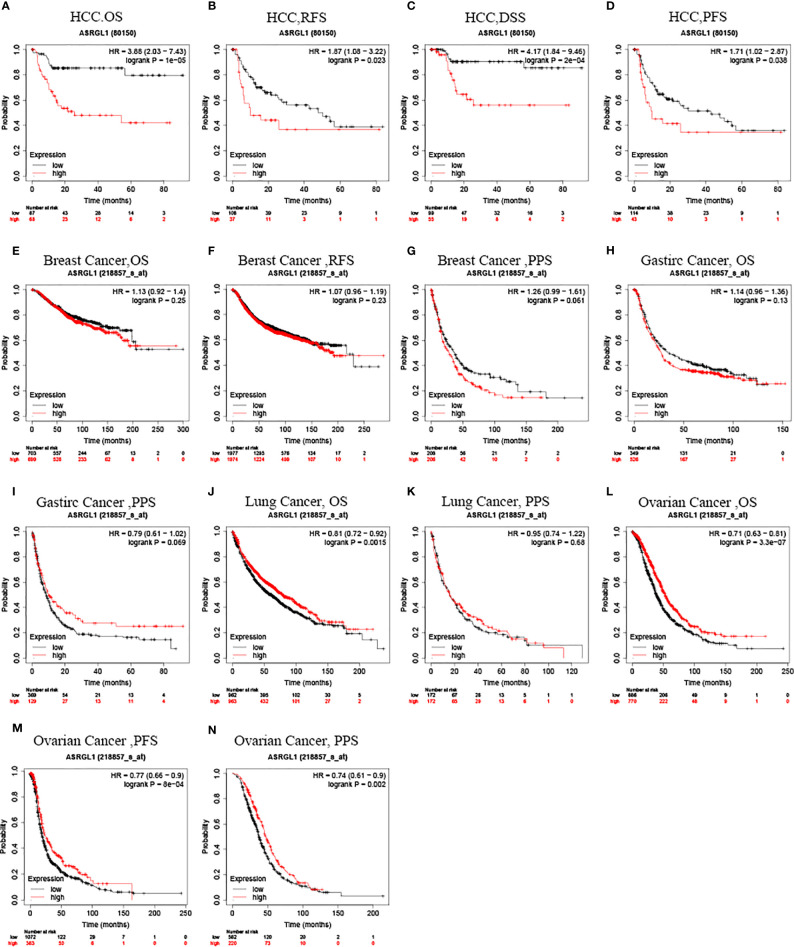
Kaplan–Meier survival curve analysis of the prognostic significance of ASRGL1 expression based on the Kaplan–Meier plotter database. **(A–D)** Asian patients with higher ASRGL1 expression showed better OS, RFS, PFS, and DSS in HCC cohorts (n = 155, n = 145, n = 157, and n = 154, respectively). **(E–G)** Survival curves of OS, RFS, and PPS in the breast cancer cohort (n = 1404, n = 3951, and n = 414, respectively). **(H, I)** OS and DFS survival curves of gastric cancer (n = 875, n = 498). **(J, K)** Survival curves of OS and PPS in the lung cancer cohort (n = 344). **(L–N)** The OS, PFS, and PPS survival curves of ovarian cancer (n = 1,656, n = 1,435, and n = 782, respectively).

Kaplan–Meier survival curves were used to identify the relationship between ASRGL1 expression and the clinical outcome of patients with cancer. The results showed that high expression of ASRGL1 was significantly correlated with poor relapse-free survival (RFS), progression-free survival (PFS), disease-specific survival (DSS), and overall survival (OS) (p < 0.05, **Figures 3A–D**) in HCC, and with overall survival (OS), progression-free survival (PFS), and post-progression survival (PPS) in ovarian cancer (**Figures 3L–N**). In addition, high ASRGL1 expression was correlated negatively with OS of patients with lung cancer (**Figure 3J**) , but not with PPS (**Figure 3K**). However, it was not associated with the outcome of patients with breast cancer or gastric cancer (**Figures 3E–I**). Analysis using the GEPIA database revealed the same outcome, indicating that high ASRGL1 expression was associated with a poor outcome (**Supplementary Figures 1A, B**). Meanwhile, the investigation using GEO dataset also produced similar results (**Supplementary Figure 1C**).

Then, we used the Kaplan–Meier plotter database to analyze the relationship between ASRGL1 expression and the clinical characteristics of patients with HCC (**Table 1**). The results showed that high expression of ASRGL1 was suggestive of poorer OS, except in patients with microvascular invasion [HR = 1.82 (0.85–3.88), P = 0.012] and that the high expression of ASRGL1 was negatively correlated with PFS in female patients and patients with stage 3, 2/3, and 3/4 cancers. In addition, high ASRGL1 expression was significantly correlated with RFS in female patients. These results suggested the prognostic significance of ASRGL1 expression and its close relationship with the clinical characteristics of HCC.

**Table 1:** Correlation of ASRGL1 expression and clinical prognosis in hepatocellular carcinoma with different clinicopathological factors by Kaplan-Meier plotter.

Clinnico pathological characteristics	Overall survial(376)			PFS(376)			RFS(376)		
	N	Hazard ration	P-value	N	Hazard ration	P-value	N	Hazard ration	P-value
**Sex**									
** female**	121	2.05(1.14-3.68)	0.014	121	1.7(1.01-2.84)	0.041	121	2(1.11-3.62)	0.019
** male**	250	2.95(1.52-5.74)	0.00081	250	1.54(1-2.38)	0.051	250	1.47(0.94-2.3)	0.093
**Stage**									
** 1**	171	1.62(0.87-3.02)	0.12	171	1.29(0.78-2.13)	0.31	171	0.78(0.45-1.35)	0.38
** 2**	86	4.67(1.1-19.87)	0.022	86	1.91(0.85-4.3)	0.11	86	1,68(0.73-3.84)	0.22
** 3**	85	2.72(1.3-5.69)	0.0058	85	2.27(1.13-4.56)	0.018	85	2.46(1.17-5.19)	0.015
** 1+2**	257	2.31(1.14-4.65)	0.016	257	1.41(0.89-2.24)	0.14	257	1.19(0.74-1.89)	0.48
** 2+3**	171	2.76(1.45-5.26)	0.0013	171	1.94(1.17-3.2)	0.0087	171	1.75(1.04-2.93)	0.032
** 3+4**	90	2.82(1.36-5.87)	0.0038	90	2.16(1.11-4.21)	0.021	90	2.46(1.17-5.19)	0.015
**Grade**									
** 1**	55	1.87(0.73-4.78)	0.18	55	1.27(0.57-2.8)	0.56	55	0.46(0.17-1.25)	0.12
** 2**	171	2.59(1.37-4.9)	0.0024	171	1.74(1.11-2.73)	0.014	171	2(1.2-3.32)	0.0065
** 3**	122	2.02(1.05-3.87)	0.031	122	1.28(0.77-2.12)	0.33	122	1.23(0.65-2.34)	0.53
**AJCC_T**									
** 1**	181	1.87(1.03-3.41)	0.038	181	1.39(0.86-2.25)	0.18	181	0.8(0.47-1.37)	0.41
** 2**	94	3.28(0.99-10.85)	0.04	94	2.06(0.97-4.39)	0.055	94	1.98(0.87-4.49)	0.095
** 3**	80	2.35(1.15-4.82)	0.016	80	1.8(0.93-3.48)	0.077	80	2(0.95-4.24)	0.064
**Vascular invasion**									
** none**	205	1.85(1.1-3.12)	0.019	205	1.24(0.8-1.94)	0.33	205	0.78(0.48-1.26)	0.31
** micro**	93	1.82(0.85-3.88)	0.12	93	0.63(0.33-1.21)	0.16	93	0.62(0.3-1.28)	0.2
**Hepatitis virus:**									
** yes**	153	2.1(1.1-4.02)	0.021	153	0.77(0.48-1.25)	0.29	153	0.69-0.41-1.15)	0.15
** none**	169	1.86(1.18-2.94)	0.0069	169	1.96(1.13-3.39)	0.015	169	1.74(0.95-3.16)	0.068

### ASRGL1 Co-Expression Network in HCC

**Figure 4:**
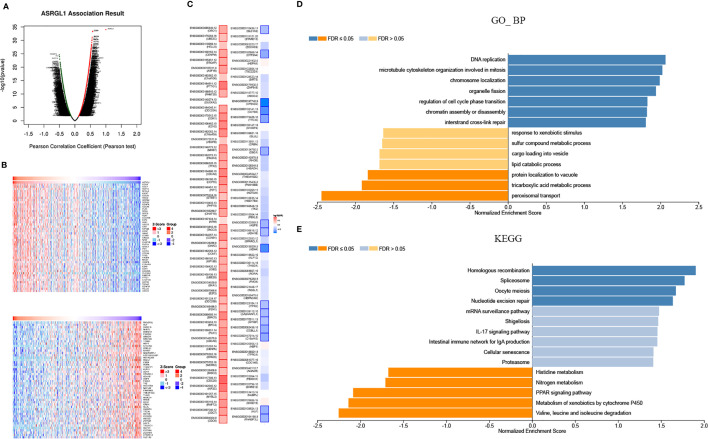
Genes co-expressed with ASRGL1 in HCC, as determined from the LinkedOmics database. **(A)** Highly correlated genes identified by the Pearson test in the HCC cohort. **(B)** The heat maps showing top 50 genes positively and negatively correlated with ASRGL1 in HCC (red: positively correlated genes; blue: negatively correlated genes). **(C)** Survival maps showing the effect of the top 50 genes positively and negatively correlated with ASRGL1 in HCC. **(D, E)** Significantly enriched GO annotations and KEGG pathways of the genes co-expressed with ASRGL1 in HCC based on LinkedOmics database.

To investigate the mechanism of action for ASRGL1, the co-expression network of ASRGL1 was constructed using the LinkedOmics database. In total, 12,786 genes were positively correlated with ASRGL1 expression, and 7,136 genes were negatively correlated (**Figure 4A**). The genes co-expressed with ASRGL1 are listed in **Supplementary Table 1**. The 50 genes with the strongest positive and negative correlation are presented in **Figure 4B**. The survival maps of the positively and negatively correlated genes are shown in **Figure 4C**. Gene set enrichment analysis (GESA) was then applied to analyze the Gene Ontology (GO) terms and Kyoto Encyclopedia of Genes and Genomes (KEGG) pathway of the genes co-expressed with ASRGL1. The GO analysis indicated that genes co-expressed with ASRGL1 were mainly involved in DNA replication and microtubule cytoskeleton organization involved in mitosis (**Figure 4D**). The KEGG pathway analysis showed that the co-expressed genes participated in spliceosome development and homologous recombination (**Figure 4E**).

### Genomic Alterations of ASRGL1 in HCC

**Figure 5:**
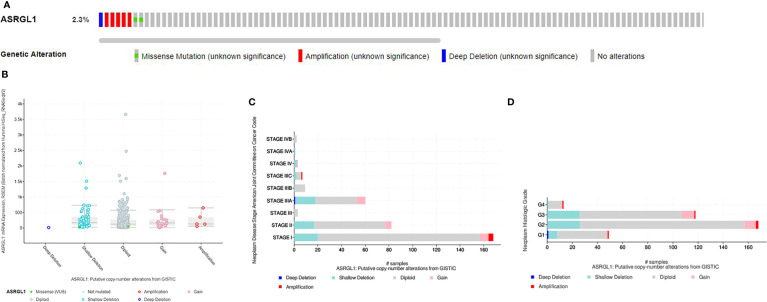
The genomic alterations of ASRGL1 in HCC as identified in cBioPortal. **(A)** OncoPrint provides an overview of the genomic alterations of ASRGL1 in HCC based on the TCGA database. **(B)** ASRGL1 expression in different ASRGL1 CNV groups. ASRGL1 amplification (AMP) group, showing a significantly higher expression level. **(C, D)** Distribution of ASRGL1 CNV frequency in different stage and grade subgroups. The percentage on the right of the bar indicates the proportion of patients with ASRGL1 gain or AMP in this subgroup.

To investigate mutations in the ASRGL1 gene in HCC, cBioPortal was used to analyze DNA sequencing data from patients with HCC. The results indicated that ASRGL1 was altered in eight (2.3%) of 370 patients with HCC (**Figure 5A**). The alterations involved mRNA missense mutation, amplification (AMP), and deep deletion; shallow deletion was the most common alteration (**Figure 5B**). Gain or AMP showed lower ASRGL1 expression levels (p < 0.001) compared with the shallow deletion. Moreover, the frequency distribution of ASRGL1 CNV in patients with different stages and grades suggested that ASRGL1 CNV alteration was an early event with a high frequency of occurrence in HCC (**Figures 5C, D**).

### Regulators of the ASRGL1 Network in HCC

**Figure 6:**
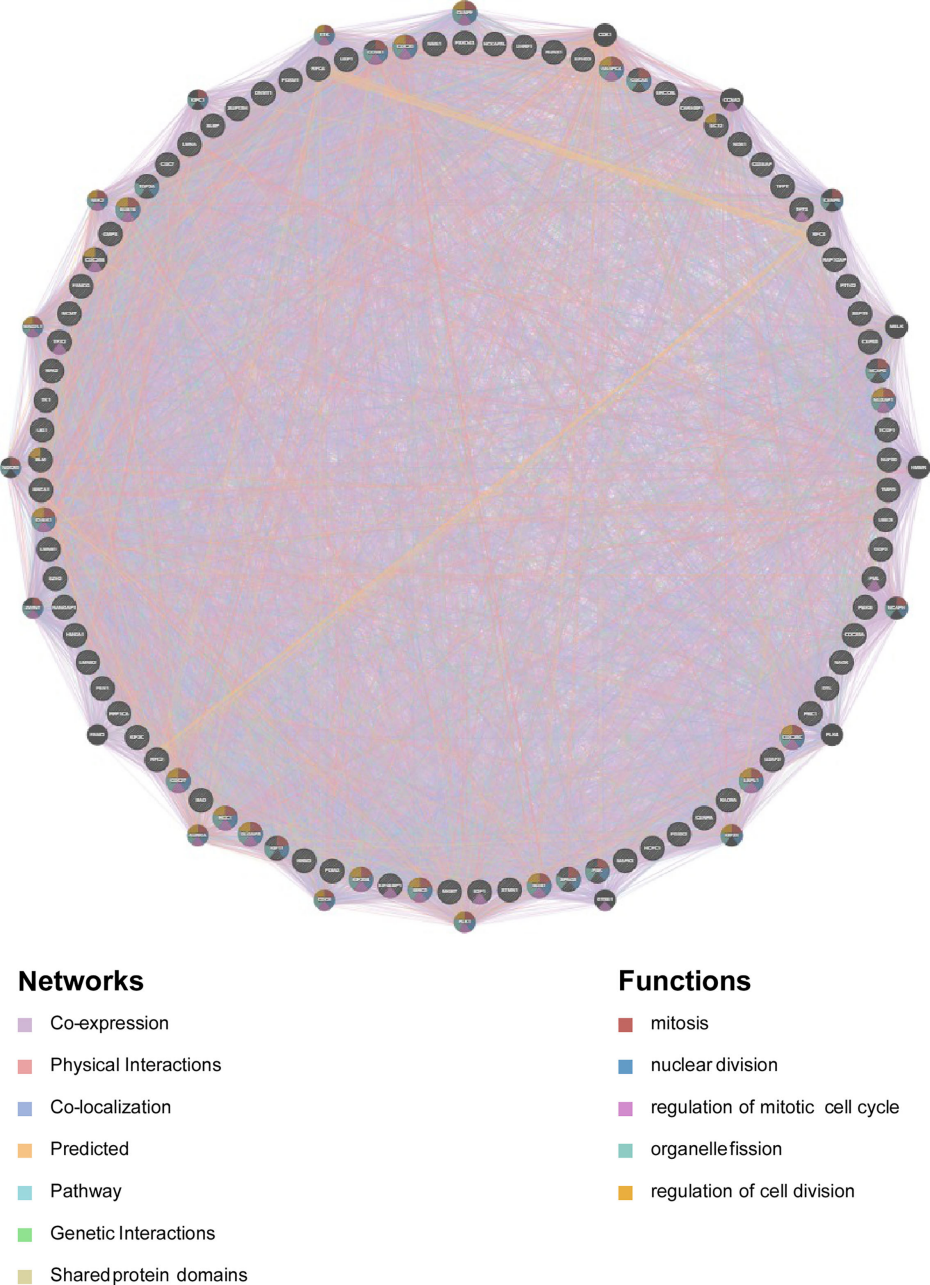
Protein–protein interaction network of CDK kinase-target networks (GeneMANIA). Protein–protein interaction (PPI) network and functional analysis indicating the gene sets enriched in the target network of ATR kinases. Different colors of the network edges indicate the bioinformatics methods applied: co-expression, website prediction, pathway, physical interactions, and co-localization. The different colors of the network nodes indicate the biological functions of the enriched gene sets.

Kinases, transcription factors, and miRNA network that may regulate ASRGL1 expression were analyzed. The five most significant kinases were CDK1, PLK1, ATR, PRKCI, and AURKA. The five most significant miRNAs related to ASRGL1 expression were MIR-296, MIR-524, MIR-34A, MIR-34C, and MIR-449. The five most significant transcription factor networks related to ASRGL1 expression were V$E2F_Q6, V$E2F_Q4, V$E2F1_Q6, V$E2F1DP2_01, and V$E2F1DP1_01. From the protein–protein interaction network constructed to investigate the main function of the genes enriched for CDK1 kinase, the gene sets enriched for CDK1 were found to be mainly involved in mitosis, nuclear division, and mitotic cell cycle regulation (**Figure 6**, **Table 2**). The functions of the gene sets enriched for transcription factor V$E2F_Q6 were DNA-dependent DNA replication and DNA replication, and DNA strand elongation involved in DNA replication.

**Table 2:** The Kinases, miRNAs and transcription factors-target networks of ASRGL1 in HCC.

Enriched Category	GeneSet	LeadingEdgeNum	FDR
**Kinase Target**	Kinase_CDK1	89	0
	Kinase_PLK1	26	0
	Kinase_ATR	25	0
	Kinase_PRKCI	11	0.004626751
	Kinase_AURKA	14	0.005397876
**miRNA Target**	V$E2F_Q6	84	0
	V$E2F_Q4	84	0
	V$E2F1_Q6	82	0
	SGCGSSAAA_V$E2F1DP2_01	63	0
	V$E2F1DP1_01	80	0
**Transcription Factor Target**	GGGGCCC,MIR-296	19	0.115147389
	CTTTGTA,MIR-524	55	0.656966871
	CACTGCC,MIR-34A,MIR-34C,MIR-449	80	1
	GAGCCAG,MIR-149	46	1
	TCCCCAC,MIR-491	15	0.642697653

### Strong Correlation Between ASRGL1 and Immune Cell Infiltration in HCC

**Figure 7:**
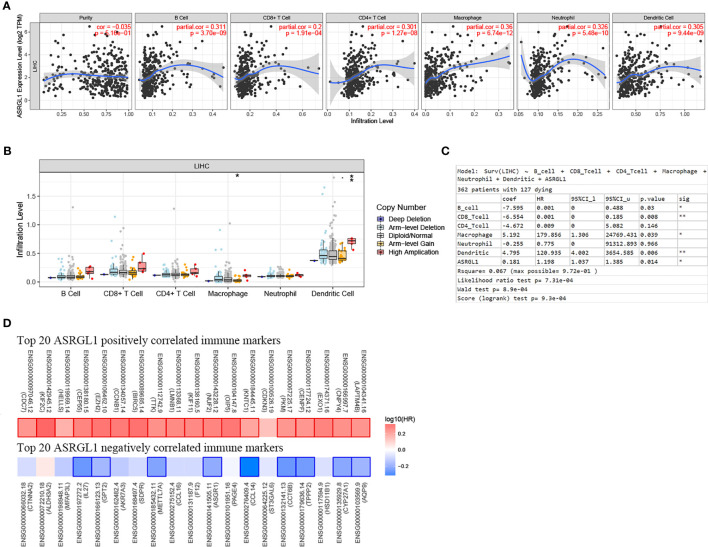
Correlation between the mRNA expression of ASRGL1 and the immune infiltration in HCC. **(A)** The significant correlation between ASRGL1 mRNA expression and infiltrating levels of B cells, CD4+ T cells, macrophages, neutrophils, and dendritic cells in HCC. **(B)** The effect of ASRGL1 CNV on the infiltrating levels of macrophages, and dendritic cells in HCC. **(C)** Evaluation of the impact of ASRGL1 expression on overall survival in the presence of infiltrating levels of multiple immune cells using multivariable hazards models. **(D)** Survival maps of the top 20 positively and negatively genes correlated with the immune markers in HCC, respectively. *p < 0.05, **p < 0.01.

Using TIMER 2.0, we found that ASRGL1 was correlated significantly with immune cell infiltration (**Figure 7A**). ASRGL1 expression showed a significant correlation with the infiltrating levels of macrophages and DCs (**Figure 7B**). Furthermore, by analyzing the effect of ASRGL1 on OS in the absence of immune infiltration cells, we found that ASRGL1 resulted in a 1.56-fold greater risk for OS of patients with HCC (**Figure 7C**). To further study the relationship between ASRGL1 and immunity, we analyzed the correlation between immune-related genes and ASRGL1. The results are shown in **Supplementary Table 2**. The survival maps of the 20 immune-related genes with the strongest positive and negative correlation with ASRGL1 in HCC are shown in **Figure 7D**.

### Association Between ASRGL1 Expression and Immune Signatures

**Figure 8:**
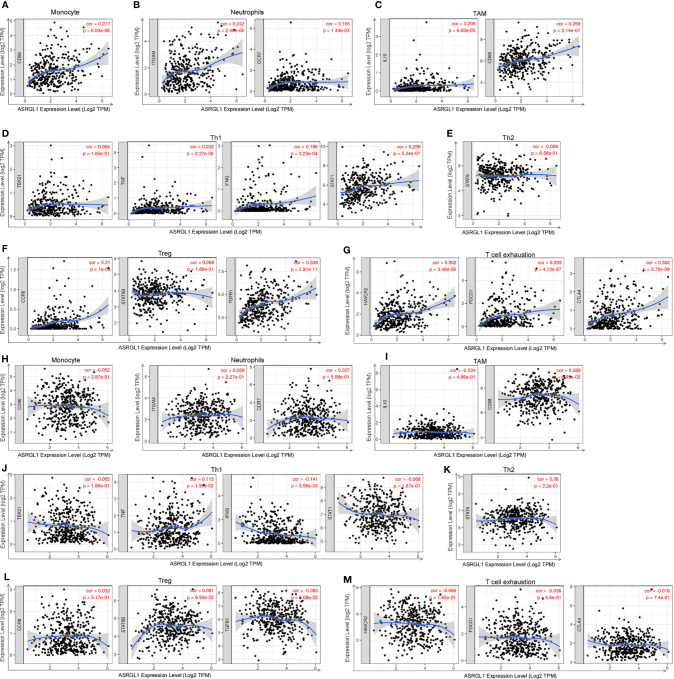
Correlation analysis of ASRGL1 expression and the expression of marker genes of infiltrating immune cells in HCC **(A–G)** and STAD **(H–M)** using the TIMER database. **(A–G)** Scatter plots showing a high correlation between the mRNA expression of ASRGL1 and the gene markers of **(A)** monocytes (CD86); **(B)** neutrophils (ITGAM and CCR7); **(C)** TAMs (IL-10 and CD68); **(D)** Th1 cells (TNF, IFNG, and STAT1); **(F)** Tregs (CCR8, STAT5B, and TGFB1); and **(G)** exhausted T cells (HAVCR2, PDCD1, and CTLA4) in HCC samples (n = 371). **(H–N)** Scatter plots showing no significant correlation between the mRNA expression of ASRGL1 and the gene markers of **(H)** monocytes (CD86); **(I)** neutrophils (ITGAM and CCR7); **(J)** TAMs (IL-10 and CD68); **(K)** Th1 cells (TBX21, IFNG and STAT1); **(L)** Th2 cells (STAT6); **(M)** Tregs (CCR8, STAT5B, and TGFB1); and **(N)** exhausted T cells (HAVCR2, PDCD1, and CTLA4) in STAD (n = 415).

To further determine the effect of ASRGL1 on TIICs, we analyzed the relationship between ASRGL1 and immune cell markers in HCC and STAD using the TIMER 2.0 database. The expression of immune cells, including CD8+ T cells, T cells (general), B cells, tumor-associated macrophages (TAMs), monocytes, neutrophils, macrophages, dendritic cells, and natural killer (NK) cells, was examined in HCC and STAD tissues. The mRNA expression of ASRGL1 was closely related to the infiltration of CD8+ T cells, T cells (general), B cells, monocyte, TAMs, macrophages, DCs, and Th1 and Treg cells in HCC tissues. In contrast, there was no significant correlation between immune cells and ASRGL1 in STAD tissues (**Table 3** and **Figure 8**).

**Table 3:** Correlation analysis between ASRGL1 and markers of immune cells in TIMER.

Description	Gene markers	HCC				STAD			
		none		purity		none		purity	
		Core	p	Core	p	Core	p	Core	p
**CD8+T cell**	CD8A	0.1566	0.0025	0.1297	0.0159	-0.1091	0.0263	-0.0977	0.0574
	CD8B	0.1664	0.0013	0.1434	0.0076	0.0011	0.9829	0.0182	0.7237
**T cell(general)**	CD3D	0.3220	0.0000	0.3189	0.0000	-0.0979	0.0462	-0.0883	0.0862
	CD3E	0.2469	0.0000	0.2529	0.0000	-0.0510	0.3001	-0.0345	0.5029
	CD2	0.2649	0.0000	0.2694	0.0000	-0.0786	0.1099	-0.0602	0.2423
**B cell**	CD19	0.3082	0.0000	0.3019	0.0000	0.1082	0.0276	0.1086	0.0345
	CD79A	0.1718	0.0009	0.1605	0.0028	0.0291	0.5544	0.0408	0.4285
**Monocyte**	CD86	0.2771	0.0000	0.2938	0.0000	-0.0524	0.2867	-0.0413	0.4229
	CD115(CSF1R)	0.1807	0.0005	0.1885	0.0004	-0.0119	0.8086	-0.0228	0.6575
**TAM**	CCL2	0.1150	0.0268	0.1146	0.0333	-0.0082	0.8683	-0.0080	0.8763
	CD68	0.2663	0.0000	0.2587	0.0000	0.0892	0.0695	0.0955	0.0633
	IL10	0.2056	0.0001	0.2053	0.0001	-0.0343	0.4864	-0.0419	0.4164
**M1 Macrophase**	INOS(NOS2)	-0.0203	0.6962	-0.0310	0.5665	0.1196	0.0148	0.0994	0.0532
	IRF5	0.3074	0.0000	0.3191	0.0000	0.0071	0.8860	0.0073	0.8873
	COX2(PTGS2)	0.1548	0.0028	0.1809	0.0007	0.0924	0.0599	0.0808	0.1164
**M2 Macrophage**	CD163	0.1043	0.0446	0.0919	0.0882	-0.0340	0.4901	-0.0365	0.4781
	VSIG4	0.1442	0.0054	0.1412	0.0086	-0.0803	0.1022	-0.0824	0.1093
	MS4A4A	0.1538	0.0030	0.1600	0.0029	-0.0805	0.1013	-0.0839	0.1030
**Neutrophils**	CD66B	0.0789	0.1292	0.0892	0.0979	0.0800	0.1035	0.0797	0.1213
	CD11B(ITGAM)	0.2424	0.0000	0.2619	0.0000	0.0594	0.2274	0.0654	0.2036
	CCR7	0.1649	0.0014	0.1870	0.0005	0.0267	0.5877	0.0376	0.4656
**Natural kill cell**	KIR2DL1	-0.0218	0.6750	-0.0590	0.2745	-0.0168	0.7334	-0.0087	0.8661
	KIR2DL3	0.0734	0.1580	0.0563	0.2975	-0.0905	0.0654	-0.0845	0.1005
	KIR2DL4	0.0956	0.0658	0.0744	0.1682	-0.0267	0.5873	-0.0127	0.8055
	KIR3DL1	-0.0345	0.5082	-0.0477	0.3775	-0.0132	0.7891	0.0018	0.9720
	KIR3DL2	0.0557	0.2847	0.0240	0.6573	-0.0588	0.2318	-0.0466	0.3658
	KIR3DL3	0.0189	0.7168	-0.0087	0.8715	0.0534	0.2776	0.0659	0.2008
	KIR2DS4	0.0383	0.4618	0.0134	0.8047	-0.0331	0.5009	-0.0276	0.5919
**Dendritic cell**	HLA-DPB1	0.1840	0.0004	0.1762	0.0010	-0.0852	0.0830	-0.0803	0.1186
	HLA-DQB1	0.1823	0.0ty -50004	0.1688	0.0017	-0.0392	0.4254	-0.0392	0.4473
	HLA-DRA	0.1905	0.0002	0.1914	0.0004	-0.0834	0.0896	-0.0732	0.1547
	HLA-DPA1	0.1720	0.0009	0.1796	0.0008	-0.0616	0.2102	-0.0509	0.3226
	BDCA-1(CD1C)	0.1606	0.0019	0.1687	0.0017	0.0653	0.1844	0.0635	0.2178
	BDCA-4(NRP1)	0.0973	0.0610	0.0959	0.0754	-0.0234	0.6339	-0.0387	0.4528
	CD11C(ITGAX)	0.3182	0.0000	0.3441	0.0000	0.0321	0.5140	0.0325	0.5279
**Th1**	T-BET(TBX21)	0.0840	0.1060	0.0720	0.1821	-0.0650	0.1861	-0.0594	0.2485
	STAT4	0.2981	0.0000	0.3035	0.0000	-0.0122	0.8042	-0.0061	0.9064
	STAT1	0.2577	0.0000	0.2423	0.0000	-0.0680	0.1668	-0.0497	0.3345
	IFN-R	0.1857	0.0003	0.1801	0.0008	-0.1410	0.0040	-0.1269	0.0135
	TNF-A(TNF)	0.2421	0.0000	0.2590	0.0000	0.1146	0.0196	0.1414	0.0058
**Th2**	GATA3	0.2293	0.0000	0.2401	0.0000	-0.1227	0.0124	-0.1152	0.0250
	STAT6	-0.0093	0.8584	-0.0102	0.8504	0.0604	0.2198	0.0720	0.1619
	STAT5B	0.0684	0.1884	0.0965	0.0735	0.0810	0.0993	0.0722	0.1608
	IL13	0.0769	0.1392	0.0859	0.1112	0.0391	0.4273	0.0501	0.3305
**Tfh**	BCL6	-0.0199	0.7021	-0.0102	0.8509	-0.0951	0.0529	-0.1006	0.0503
	IL21	0.0518	0.3198	0.0543	0.3145	-0.0528	0.2836	-0.0416	0.4191
**Th17**	STAT3	0.2202	0.0000	0.2237	0.0000	0.1111	0.0236	0.1050	0.0411
	IL17A	-0.0247	0.6356	-0.0092	0.8655	0.1625	0.0009	0.1667	0.0011

Cor, R value of Spearman’s correlation; None, correlation without adjustment. Purity, correlation adjusted by purity.*P < 0.05, **P < 0.01, ***P < 0.001.

The correlation between immune cells and ASRGL1 was also analyzed using the GEPIA database. The mRNA expression of ASRGL1 was positively correlated with monocytes, neutrophils, TAM, Th1, Treg, and T cell exhaustion, but not with Th2 cell infiltration, whereas the correlation between immune cells and ASRGL1 was not significant in STAD tissues (**Table 4**). All results indicated that ASRGL1 may participate widely in tumor immune cell infiltration and have a vital role in the occurrence of HCC.

**Table 4:** Correlation analysis between ASRGL1 and markers of immune cells in GEPIA.

Description	Gene markers	HCC				STAD			
		Tumor		Normal		Tumor		Normal	
		R	P	R	P	R	P	R	P
**Monocyte**	**CD86**	0.21	3.30E-05	0.18	0.21	-0.086	0.18	0.57	0.00032
**Neutrophils**	**CD11b**	0.29	1.90E-08	0.16	0.26	0.047	0.34	0.27	0.11
	**CCR7**	0.0051	0.92	0.14	0.33	-0.031	0.53	0.4	0.016
**TAM**	**CD68**	0.22	1.70E-05	0.28	0.047	0.018	0.72	0.16	0.35
	**IL-10**	0.043	0.41	0.0093	0.95	0.11	0.027	0.4	0.016
**Th1**	**IFN-γ(IFNG)**	0.11	0.029	0.064	0.66	-0.13	0.0072	0.37	0.028
	**STAT1**	0.16	0.026	0.27	0.054	-0.056	0.26	0.33	0.047
	**T-bet(TBX21)**	0.025	0.64	0.091	0.54	-0.12	0.017	0.62	4.90E-05
	**TNF-α(TNF)**	0.017	0.75	0.0082	0.96	0.1	0.041	0.14	0.43
**Th2**	**STAT6**	0.065	0.21	0.1	0.48	0.061	0.22	-0.17	0.31
**Treg**	**CCR8**	0.31	1.50E-09	0.2	0.17	-0.017	0.73	0.18	0.29
	**STAT5B**	0.054	0.3	-0.02	0.89	0.021	0.67	-0.027	0.11
	**TGF-β(TGFB1)**	0.17	0.0013	0.23	0.11	-0.073	0.14	-0.3	0.078
**T cell exhaustion**	**CTLA4**	0.25	1.60E-06	0.19	0.18	0.079	0.11	0.43	0.0095
	**PD-1(PDCD1)**	0.074	0.15	0.12	0.4	-0.032	0.52	0.5	0.0018
	**TIM-3(HAVCR2)**	0.1	0.054	0.28	0.052	-0.054	0.28	0.58	0.00019

## Discussion

As the progression of HCC is rapid, patients usually have a poor prognosis; the 5-year survival rate is below 10% (21). Therefore, it is of great significance to explore the pathogenesis of HCC and identify potential target molecules for treatment. The enzyme ASRGL1 catalyzes asparagine synthesis (22), and many studies have shown that ASRGL1 is abnormally expressed in tumors. For example, ASRGL1 is highly expressed in breast carcinoma compared with the adjacent tissues, suggesting it can serve as a biomarker for cancer diagnosis (23). Tumor cells cannot synthesize asparagine, an amino acid necessary for growth, and must rely on host supply. This product can make asparagine hydrolyze, so that tumor cells lack asparagine, thereby inhibiting the growth of the role. However, the specific expression pattern and function of ASRGL1 in liver are still unclear, but it has been reported that Asn can inhibit tumor proliferation, invasion, and migration in liver cancer (24). Asparaginase can antagonize ASRGL1 in the synthesis of asparagine. In our study, it was found that ASRGL1 was highly expressed in liver cancer, which may play a role in promoting tumor development through the antagonism of asparaginase and lead to poor prognosis of patients.

In this study, through the analysis using the HCCDB and Oncomine databases, we found that ASRGL1 expression was significantly altered in HCC. To verify the above results, we examined the clinical tissue specimens of HCC and found that ASRGL1 expression was significantly higher in tumor tissues than the adjacent tissues. Meanwhile, the investigation of liver cancer cells revealed that ASRGL1 protein expression was significantly higher than that in LO2 healthy liver cells. However, we found that the expression of ASRGL1 in L02 was higher than that in Huh7 cell line. At present, some literature also showed that the expression of some oncogenes, such as DDK1 and KLF8, was increased in normal liver cell line (25, 26); the specific reasons for the higher expression of ASRGL1 in L02 than that in Huh7 are still unclear and need to be further studied. These results indicated that ASRGL1 may have an important role in the development of HCC and could be used as a tumor biomarker.

The analysis of the UALCAN database indicated that the expression of ASRGL1 in HCC tissues also varied in different clinical tumor stages, with a significant positive correlation between ASRGL1 expression and the degree of tumor malignancy. The analysis of the KP and GEPIA databases revealed a significant negative correlation between ASRGL1 expression and patient prognosis, which was verified by the GEO dataset. Patients with higher ASRGL1 expression had a poorer prognosis than patients with lower expression. These results suggested that ASRGL1 may participate in the occurrence and progression of the tumor and may be a biomarker for the early diagnosis of HCC.

To identify the regulators of ASTGL1 expression, we constructed a co-expression network, which indicated that a network of kinases, including CDK1, PLK1, ATR, PRKCI, and AURKA, may participate in the development of HCC. CDK1 is an oncogene that can promote the development of HCC (27). A variety of CDK1 inhibitors have been developed, and some have entered clinical trials for cancer treatment (28).

From the database analysis, we found that ASRGL1 was extensively involved in tumor immune invasion. A close relationship has been demonstrated between tumor occurrence and the uncontrolled immune regulation of tumors (29). The inactivation of tumor T cells, a common immune failure in liver cancer (30), can promote tumor occurrence. Recently, using single-cell sequencing technology, a subset of T cells has been found to participate in the development of the HCC (31). In this study, we found that the mRNA expression of ASRGL1 was strongly correlated with T cell infiltration, including CD4+ cells, CD8+ cells, and Treg cells. The loss of CD4+T cells was found to promote HCC development (32). The exhaustion of CD8+T cells was significantly correlated with the expression of the marker PD-1, a checkpoint of HCC (33). A high density of tumor-infiltrating B cells indicated a promising outcome for patients (34). In the present study, we found a strong correlation between ASRGL1 expression and B cell infiltration, which indicated that the ASRGL1 may participate in the development of HCC through immune infiltration.

## Conclusion

Our study has demonstrated the biological function and expression of ASRGL1 in HCC using bioinformatics analyses, and we verified these results in tissue samples. These results showed that ASRGL1 was overexpressed in HCC and had a significant negative correlation with the outcome of patients with HCC, suggesting that ASRGL1 is a biomarker for HCC. Moreover, our results also indicate the potential role of ASRGL1 in regulating immune cell infiltration. However, these findings need to be verified by large-scale genomics research and molecular mechanism studies for HCC.

## Data Availability Statement

The original contributions presented in the study are included in the article/**Supplementary Material**. Further inquiries can be directed to the corresponding authors.

## Ethics Statement

The studies involving human participants were reviewed and approved by the Affiliated Changzhou No. 2 People’s Hospital of Nanjing Medical University. The patients/participants provided their written informed consent to participate in this study.

## Author Contributions

XQ and CZ conceived and designed the study. CX and PG collected the human tissues and conducted the experiments. CX, XZ, PG, XC, LJ, and RL performed data mining. CX wrote the manuscript. All authors contributed to the article and approved the submitted version.

## Funding

This study was supported by grants from the National Natural Science Foundation of China (grant no. 81672469), The Social Development Foundation of Science and Technology of Jiangsu (grant no. BE2016658), and the project of Changzhou medical innovation team (CCX201807).

## Conflict of Interest

The authors declare that the research was conducted in the absence of any commercial or financial relationships that could be construed as a potential conflict of interest.

## Supplementary Material

Supplementary Table 1 The co-expressed genes of ASRGL1

Supplementary Table 2 Correlation of ASRGL1 with various immune signatures.

## Abbreviations

HCC, hepatocellular carcinoma; STAD, stomach adenocarcinoma; OS, overall survival; RFS, relapse-free survival; PFS, progression-free survival; GO, gene oncology; KEGG: Kyoto Encyclopedia of Genes and Genomes.
