# Supplementary figures and images for: ASRGL1 Correlates With Immune Cell Infiltration in Hepatocellular Carcinoma and Can Serve as a Prognostic Biomarker
Source: Front Oncol. 2021 Jun 25;11:680070. doi: 10.3389/fonc.2021.680070 (PMC8267417; doi:10.3389/fonc.2021.680070)

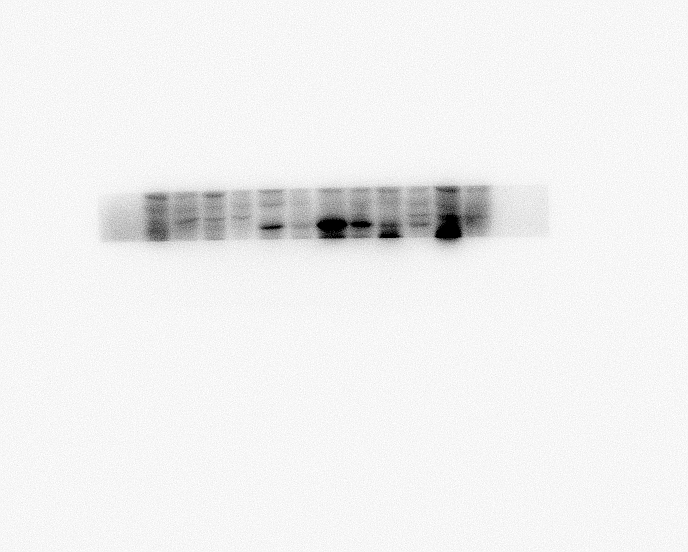

Supplement: Supplementary file 3 [file DataSheet_1.zip › Western blot/ASRGL1-HCC1.tif]

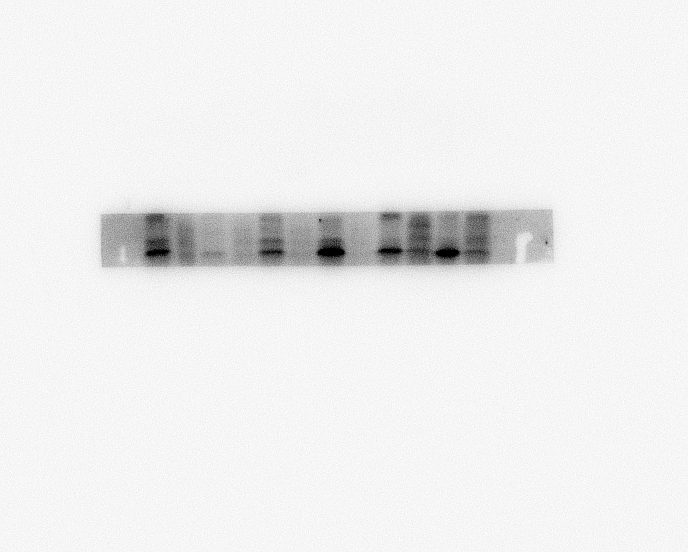

Supplement: Supplementary file 3 [file DataSheet_1.zip › Western blot/ASRGL1-HCC2.tif]

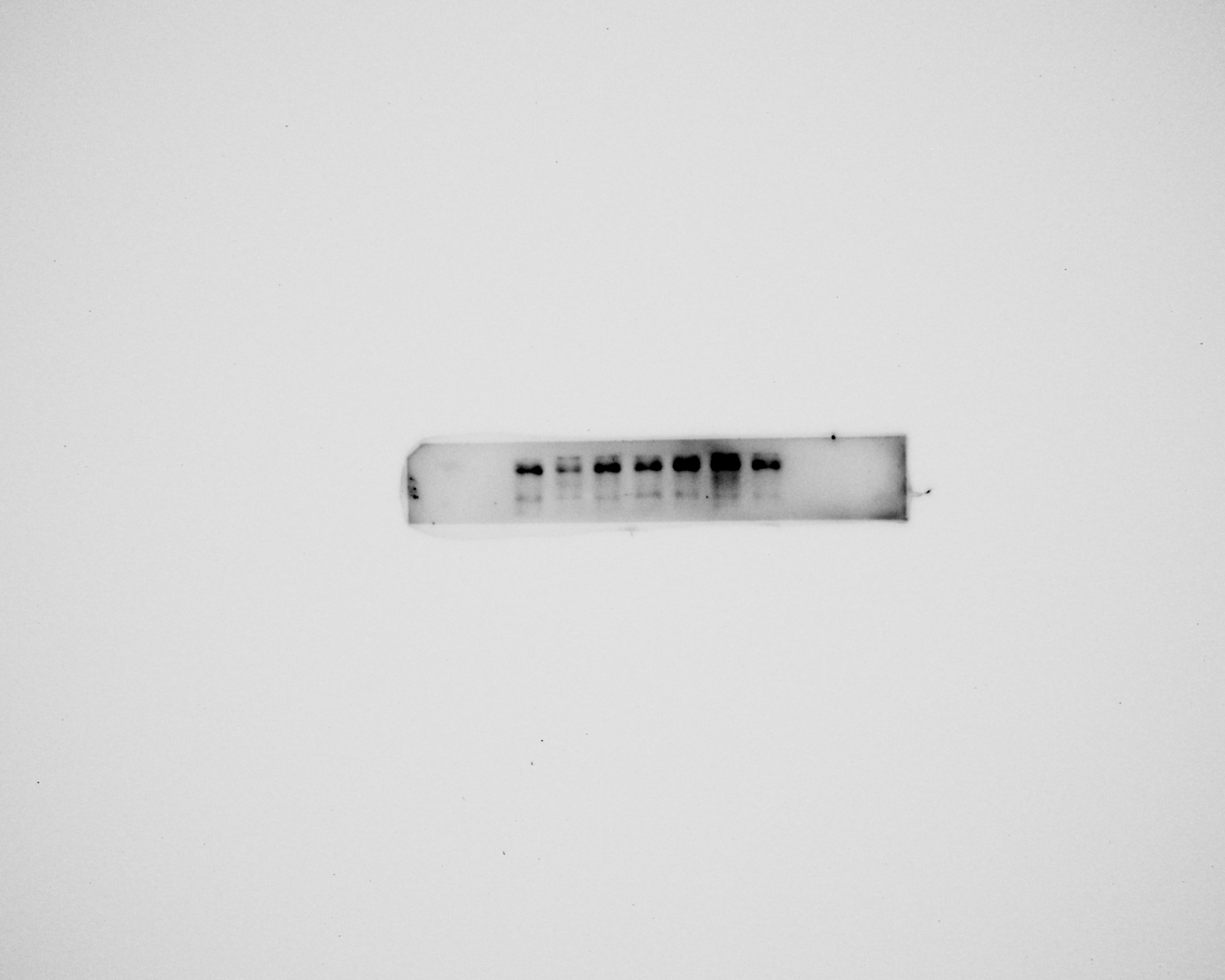

Supplement: Supplementary file 3 [file DataSheet_1.zip › Western blot/ASRGL1-NO.2.tif]

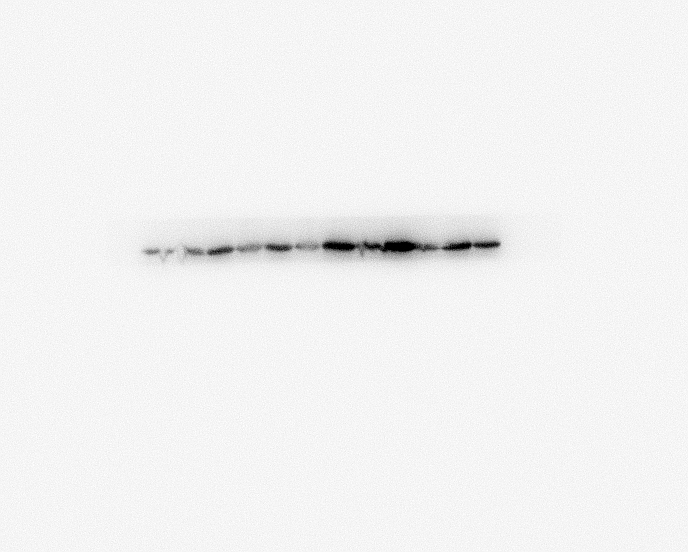

Supplement: Supplementary file 3 [file DataSheet_1.zip › Western blot/GAPDH-HCC1.tif]

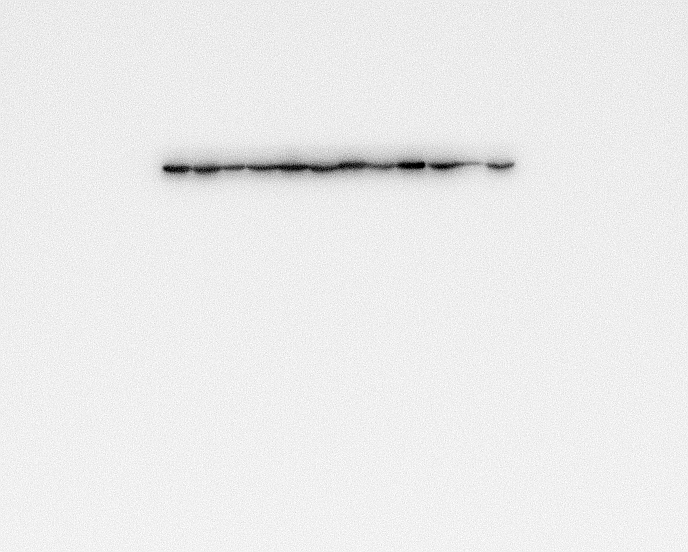

Supplement: Supplementary file 3 [file DataSheet_1.zip › Western blot/GAPDH-HCC2.tif]

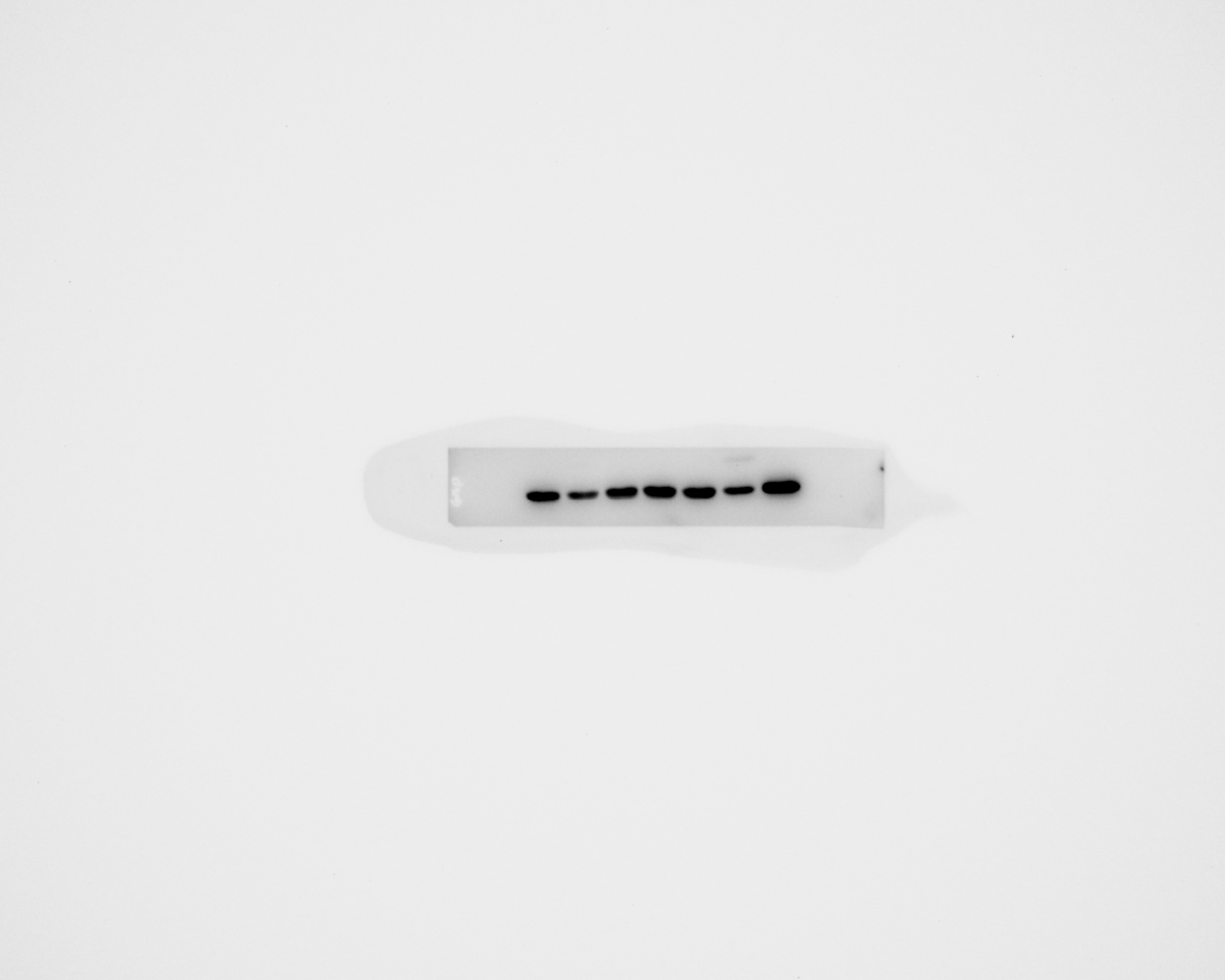

Supplement: Supplementary file 3 [file DataSheet_1.zip › Western blot/GAPDH-NO.2.tif]

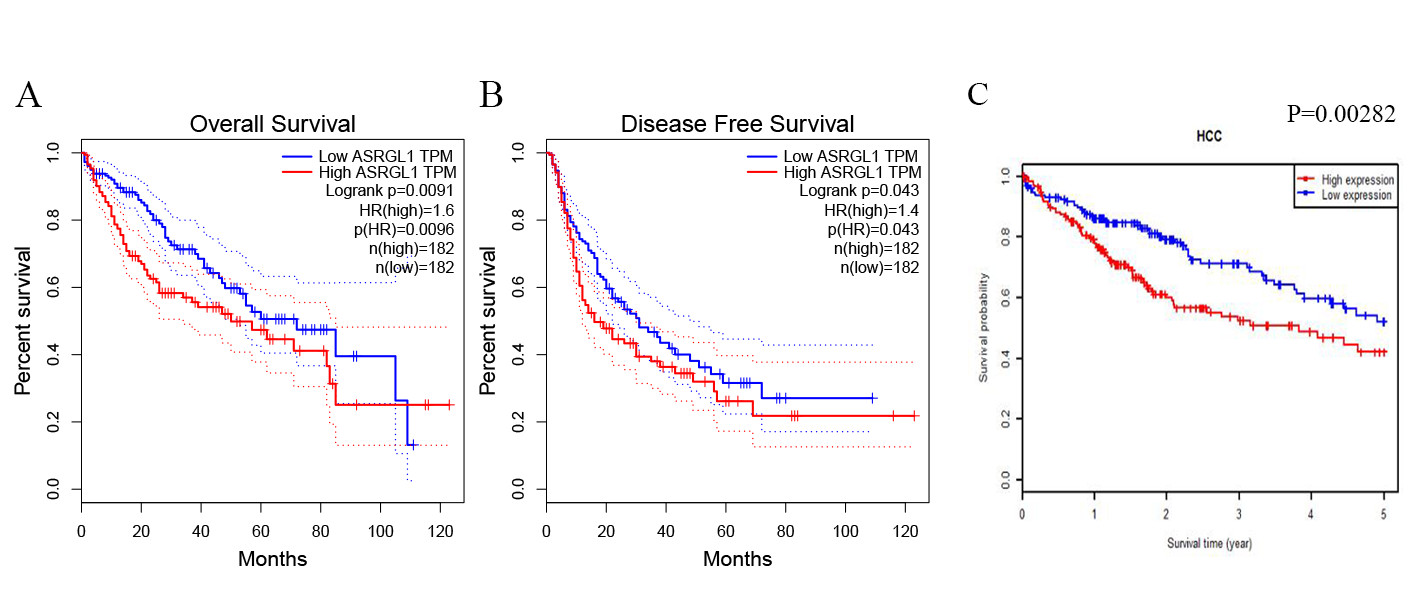

Supplement: Supplementary file 4 [file Image_1.tif]
